# Diverse Biological Functions and Domain‐Specific Interactions of the Diguanylate Cyclase PA2072 in *Pseudomonas aeruginosa* PAO1

**DOI:** 10.1155/ijm/7292171

**Published:** 2026-07-18

**Authors:** Xiaojing Gao, Yan Zhang, Lingna Guo, Huimin Zhang, Jianwen Sun, Jianfang Sun, Weidong Huang

**Affiliations:** ^1^ School of Basic Medicine, Ningxia Medical University, Yinchuan, China, nxmu.edu.cn; ^2^ Tianjin Key Laboratory of Function and Application of Biological Macromolecular Structures, School of Life Sciences, Tianjin University, Tianjin, China, tju.edu.cn; ^3^ Medical Sci-Tech Research Center, Ningxia Medical University, Yinchuan, China, nxmu.edu.cn; ^4^ Medical Science Research Institution of Ningxia Hui Autonomous Region, Yinchuan, China; ^5^ Department of Biochemistry and Molecular Biology, School of Basic Medicine, Yinchuan Ningxia Medical University, Yinchuan, China; ^6^ Department of Geriatrics, PLA 942 Hospital, Yinchuan, China

**Keywords:** bacterial two-hybrid, c-di-GMP, diguanylate cyclase, PA2072, *Pseudomonas aeruginosa*

## Abstract

Nucleotide bis‐(3,5)‐cyclic di‐guanosine monophosphate (c‐di‐GMP) is a universal and versatile secondary messenger. It is involved in the regulation of significant functions such as motility, biofilm formation, virulence, drug resistance, cell cycle, differentiation, and many other physiological processes in bacteria. In this study, we focused on the multidomain c‐di‐GMP metabolizing protein PA2072 from *Pseudomonas aeruginosa* PAO1. Phenotypic observations, transcriptional assays, and bacterial two‐hybrid analyses were used to characterize the functions of PA2072. *P. aeruginosa* PAO1 PA2072 mainly acted as c‐di‐GMP diguanylate cyclase. Its N‐terminal CHASE4 domain is largely related to siderophore production, and its C‐terminal catalytic GGDEF–EAL domain is linked to motility, in accordance with the transcriptome analysis. Subcellular observations revealed that PA2072 is a bipolar‐localized protein. The N‐terminal CHASE4 domain and C‐terminal GGDEF–EAL domain display different protein–protein interaction patterns with other c‐di‐GMP metabolizing proteins. The complicated physiological functions of c‐di‐GMP metabolizing proteins may be related to their multidomain architectures. Protein–protein interactions among c‐di‐GMP metabolizing proteins likely contribute to functional differentiation and complexity. Our work may thus help to add data and to deepen our understanding in the field of c‐di‐GMP signaling.

## 1. Introduction

In bacteria, bis‐(3,5)‐cyclic di‐guanosine monophosphate (c‐di‐GMP) was first identified as an allosteric regulator of cellulose synthase in *Gluconacetobacter xylinus* [1]. Researchers soon found that c‐di‐GMP is a universal secondary messenger that plays significant regulatory roles in bacterial motility, biofilm formation, virulence, drug resistance, the cell cycle, differentiation, and many other physiological processes [2]. c‐di‐GMP is synthesized by diguanylate cyclases (DGCs) with GGDEF (Gly‐Gly‐Asp‐Glu‐Phe) domains [3] and is degraded by c‐di‐GMP‐specific phosphodiesterases (PDEs) with EAL (Glu‐Ala‐Leu) or HD‐GYP domains [4, 5], respectively. These GGDEF, EAL, and HD‐GYP domain‐containing proteins are thus known as c‐di‐GMP metabolizing proteins.

Notably, multiple copies of c‐di‐GMP metabolizing proteins are encoded in bacterial genomes (https://www.ncbi.nlm.nih.gov/Complete_Genomes/c-di-GMP.html) [2]. In contrast, deletions of individual DGCs and PDEs are generally associated with highly specific knockout phenotypes. This raises the question of how, as a water‐soluble, free‐diffusing nucleotide, c‐di‐GMP could achieve signaling specificity [6].

With the burst of c‐di‐GMP studies, it has been recognized that both global [7] and local [8] signaling modes exist, contributing to c‐di‐GMP signaling specificity [9]. Intriguingly, in the local signaling scenario, protein–protein interactions (PPIs) are highly involved and play pivotal roles: In the simple mode, a particular DGC and/or PDE directly teams up with a specific effector/target [10]; in the more complicated mode, represented by the PdeR–DgcM–MlrA system [11] in *Escherichia coli* and the Lap system in pseudomonads [12], a number of GGDEF and EAL domain proteins interact with their effectors and also with other c‐di‐GMP metabolizing proteins, forming a signaling network.

Interestingly, c‐di‐GMP metabolizing proteins usually possess complicated molecular architectures: The catalytic GGDEF and EAL domains are located at the C‐terminus, whereas the structurally and functionally diversified sensory domains are associated with the N‐terminus [13]. It has long been observed that the C‐terminal DGC and/or PDE enzymatic activities are altered as the N‐terminal sensory domains respond to environmental physical and chemical factors [14]; however, the exact functions of the individual domains in terms of PPI are largely unknown.

Bacterial two‐hybrid (BTH) system (Euromedex BACTH system, for “Bacterial Adenylate Cyclase–based Two‐Hybrid”) is a straightforward and convenient method for in vivo PPI analysis [15, 16]. In this study, we focused on *Pseudomonas aeruginosa* PAO1, whose genome has been fully sequenced and annotated. In total, the *P. aeruginosa* PAO1 genome contains 42 c‐di‐GMP metabolizing proteins, indicating a complicated c‐di‐GMP signaling network. One of the multidomain c‐di‐GMP metabolizing proteins, PA2072, was selected for further analysis. We found that PA2072 may act as a DGC and is involved in diverse physiological functions. PPI patterns of different domains in PA2072 are diversified. Hopefully, our study provides more data and deepens our understanding of c‐di‐GMP signaling.

## 2. Materials and Methods

### 2.1. Bacterial Strains, Media, and Growth Conditions

The strains used in this study are listed in Supporting Information 2: Table S1. *P. aeruginosa* and *E. coli* strains were cultured in Luria–Bertani (LB) medium (10 g/L tryptone, 10 g/L NaCl, and 5 g/L yeast extract). Antibiotics were used at the following concentrations: 5 *μ*g/mL tetracycline (TET), 100 *μ*g/mL carbenicillin (CAR), and 50–300 *μ*g/mL gentamycin (GEN) for *P. aeruginosa* and 100 *μ*g/mL ampicillin (AMP) and 50 *μ*g/mL kanamycin (KAN) for *E. coli*.

### 2.2. Generation of Mutant Strains

*P. aeruginosa* PAO1 in‐frame deletion mutants (full‐length *pa2072*, *pa2072chase4*, or *pa2072ggdef-eal*) were generated. Flanking regions of the target DNA were amplified, fused into the pEX18Gm vector, and then introduced into PAO1 by electroporation at a condition of 2.5 kV with a decaying exponential waveform (25 *μ*F capacitor, 600 *Ω*) in a 0.2 cm cuvette. The transformants were selected on LB plates containing GEN (300 *μ*g/mL). Single colonies underwent sucrose (10%) counterselection on LB agar plates. Deletions were confirmed in candidate mutants by PCR and sequencing. For complementation, the DNA fragment of PA2072 was amplified by PCR and cloned into the pUCP20 vector. The resultant plasmid was transformed into *P. aeruginosa* mutant strains and selected on LB agar plates containing CAR (100 *μ*g/mL).

### 2.3. Motility Analysis

Overnight cultures of *P. aeruginosa* (OD_600_ = 0.6, diluted 1:100) were spot‐inoculated onto motility plates and incubated at 37°C for 12 h for swimming motility and 24 h for twitching motility. Swimming motility plates consisted of 1% tryptone, 0.5% NaCl, and 0.3% agarose, whereas twitching motility plates contained 1% tryptone, 0.5% NaCl, 0.5% yeast extract, and 1% agar.

### 2.4. Siderophore Analysis

Chrome azurol sulfonate (CAS) assay: Overnight cultures (OD_600_ = 0.6) were spotted on LB agar plates containing 1× CAS test solution (Coolaber, Beijing, China) and incubated for 24 h at 37°C. Siderophore production was quantified by measuring absorbance at 630 nm wavelength and calculated as follows: [(*A*_r_ − *A*_s_)/*A*_r_] × 100*%* (*A*_r_: absorbance of the reference, *A*_s_: absorbance of the sample).

Pyochelin extraction: The supernatant from 24‐h cultures of *P. aeruginosa* grown in succinate synthetic medium (SSM; K_2_HPO_4_ 6 g/L, KH_2_PO_4_ 3 g/L, (NH_4_)_2_SO_4_ 1 g/L, MgSO_4_ 0.1 g/L, and succinic acid 4 g/L) was acidified with citric acid and extracted with ethyl acetate. The extract was filtered through a syringe filter containing anhydrous magnesium sulfate and stored at −20°C. Absorbance was measured at a 320 nm wavelength.

### 2.5. Determination of the Relative c‐di‐GMP Levels In Vivo

Intracellular c‐di‐GMP levels were quantified using a transcriptional reporter plasmid (p*CdrA*‐GFP; kindly provided by Dr. Rice Scott, Nanyang Technological University). The plasmid was introduced into *P. aeruginosa* strains via electroporation [17]. Overnight cultures (LB containing GEN 25 *μ*g/mL) were diluted 200‐fold and grown for 24 h. The cells were harvested, washed, and resuspended in PBS. GFP fluorescence was measured using a Synergy H1 microplate reader (BioTek, United States) with 485 nm excitation and 520 nm emission filters. Relative fluorescence units (RFUs) were calculated as arbitrary fluorescence intensities normalized to cell density (OD_600_).

### 2.6. Congo Red (CR) Plate Assay

Overnight LB cultures (OD_600_ = 1.0) were spotted (1 *μ*L aliquots) onto CR (Sigma, United States) indicator plates: M9 minimal agar (0.2% glucose, 20 *μ*g/mL CR, and 0.2 mM IPTG) for *E. coli* strains, incubated at 30°C for 14 days; LB agar (40 *μ*g/mL CR and 20 *μ*g/mL Coomassie Brilliant Blue G‐250) for *P. aeruginosa* strains, incubated at 37°C for 48 h.

### 2.7. Determination of the Intracellular c‐di‐GMP Level Using LC‐MS

Overnight cultures of individual strains were inoculated into liquid LB and amplified. Cells from 1 mL of the bacterial culture (~OD_600_ = 1.8) were harvested by centrifugation and washed twice with cold PBS. Pellets were resuspended in 100 *μ*L of precooled extraction buffer (a mixture of 40% methanol, 40% acetonitrile, and 20% distilled water), incubated on ice for 15 min, and boiled at 100°C for 10 min. Centrifuge the sample at 14,000 rpm, 4°C for 10 min. Repeated the extraction for two times.

The supernatants were lyophilized and reconstituted in ultrapure water for LC‐MS analysis. The pellets were applied for total protein concentration determination using the bicinchoninic acid (BCA) method, with BSA as the standard. Absorbance was measured at 562 nm, and concentrations were calculated from a standard curve. A liquid chromatography–mass spectrometry (AB Sciex Triple Quad 4500 System, United States) was used to measure the c‐di‐GMP. The sample was dissolved in 120 *μ*L ultrapure water. Ten microliters of each sample was taken for loading. The separation of c‐di‐GMP was performed using a C18 column (Phenomenex Synergi 4 *μ*m Fusion‐RP 80 Å, 50 × 2 mm column, United States). Phase A is 0.1% formic acid:water (V/V), and phase B is 0.1% methanol:acetonitrile (V/V). Flow rate was at 0.24 mL/min with column temperature of 40°C.

c‐di‐GMP was detected using negative ion mode with an ionization voltage of 5.5 kV. The mass‐to‐charge ratio of c‐di‐GMP primary mass spectrum is 689.1, and that of the secondary mass spectrum is 343.9. The sample peaked at around 1.19 min. The standard curves for c‐di‐GMP (Sigma, United States) were prepared at 0.01, 0.1, 0.5, 0.8, and 1 *μ*M. Repeated the experiment three times.

### 2.8. Protein Sample Preparation

DNA fragments encoding PA2072 Per‐Arnt‐Sim (PAS)–GGDEF–EAL (300–864 aa), GGDEF–EAL (414–864 aa), GGDEF (414–587 aa), and EAL (595–864 aa) domains were PCR‐amplified from *P. aeruginosa* genomic DNA and cloned into pETM (a pET‐32a‐derived vector) with N‐terminal 6×His tags. Plasmids were transformed into *E. coli* BL21(DE3), and the recombinant protein was induced with 0.05 mM IPTG at 20°C. Proteins were purified using Ni‐NTA affinity and size selection and assessed by SDS‐PAGE (10% or 12% gels).

### 2.9. Thiazole Orange–Derived Fluorometric Assay

Substrates of GTP or c‐di‐GMP were incubated with 4 *μ*M of each PA2072 protein at 37°C and 100 rpm for 1 h (under the buffer condition of 10 mM CaCl_2_, 200 *μ*M GTP or c‐di‐GMP, 100 mM NaCl, 5% glycerol, and 20 mM Tris‐HCl, pH 7.6). The reactions were heated at 95°C for 10 min to denature the protein, which was removed by centrifugation. The supernatants were heated and centrifuged to remove the PA2072 proteins. Two hundred microliters of each supernatant was transferred to a new plate and incubated overnight at 4°C with 30 *μ*M thiazole orange in the dark. Fluorescence was measured at 20°C using an EnSpire reader (excitation 508 nm, emission 533 nm).

### 2.10. Cellular Localization by Confocal Microscopy

The full‐length *pa2072* gene fused to eYFP in the pUCP20 vector was expressed in *P. aeruginosa Δpa2072*. Overnight cultures (OD_600_ = 0.8) were harvested, washed once with 1× PBS, and subjected to cellular localization analysis using a confocal fluorescent microscope (Zeiss, LSM900, Germany; excitation 488 nm, emission 509 nm) with a 63× oil‐immersion objective and 4× digital zoom.

### 2.11. RNA‐Seq and Quantitative Real‐Time PCR Analysis

RNA‐seq was performed by Gene‐van Biotechnology Service Co. Ltd. (Beijing, China). Total RNA of 12 samples, representing four experimental groups with three independent biological replicates each, was subjected to transcriptome sequencing on the HiSeq/MGI platform, generating approximately 40 million paired‐end reads per sample. Differential expression analysis used DESeq2 with significance thresholds of FDR‐adjusted *p* value (*p* adj) ≤ 0.05 and |log2 (*f* *o* *l* *d* *c* *h* *a* *n* *g* *e*)| ≥ 1.

For real‐time PCR, total RNA was isolated using TRIzol reagent (Invitrogen, United States). Reverse transcription was performed using the SuperScript IV First‐Strand Synthesis System (Invitrogen, United States). Amplification (Takara, TB Green Premix Ex Taq II, Japan) was performed using gene‐specific primers (Supporting Information 3: Table S2) in a CFX96 Real‐Time PCR System (Bio‐Rad, United States). Gene expression was quantified using the 2^−*ΔΔ*CT^ method, with *rpoD* as the endogenous reference.

### 2.12. BTH Assay of PPIs

PPIs were assessed using the BTH system (Euromedex, BACTH System Kit, France) following established methods [18]. Plasmids encoding the target proteins fused with either the T18 fragment (pUT18C vector) or the T25 fragment (pKT25/pKNT25 vector) were cotransformed into *E. coli* BTH101 cells. Colony color was observed and recorded. For colorimetric analysis, X‐gal (Solarbio, Beijing, China) agar plates and MacConkey (Sigma, United States) agar plates containing AMP (100 *μ*g/mL) and KAN (50 *μ*g/mL) were used.

### 2.13. Protein–Protein Pull‐Down Assay

For the PA2870 and PA2072 pull‐down assay, the full‐length coding region of PA2870 was cloned into the pETM vector and expressed in *E. coli* BL21(DE3) (0.1 mM IPTG, 25°C, 20 h). The recombinant protein was purified on the Ni‐NTA resin. PA2072‐eYFP, PA2072chase4‐eYFP, and PA2072ggdef‐eal‐eYFP were cloned and expressed in *P. aeruginosa Δpa2072* from pUCP20 vectors. Lysates of these eYFP‐tagged samples were prepared by sonication in lysis buffer (Beyotime Biotechnology, China, containing 0.01% Triton and protease inhibitors) and centrifuged. The supernatant was incubated with PA2870‐bound Ni‐NTA resin for 2 h at 4°C. After washing (10 mM Tris/Cl pH 7.5, 150 mM NaCl, and 0.5 mM EDTA), bound proteins were eluted and analyzed by western blotting using anti‐GFP antibody (Abcam, United Kingdom, diluted 1:10,000).

For the PA2072 and PA1851 pull‐down assay, the GFP‐Trap magnetic beads (Proteintech, United States) were incubated with the cell lysates containing the PA2072 eYFP‐tagged proteins for 2 h at 4°C, then washed with wash buffer (10 mM Tris/Cl pH 7.5, 150 mM NaCl, 0.05% Nonidet P40 Substitute, and 0.5 mM EDTA). Full‐length PA1851‐mCherry was cloned into the PME6032 vector and expressed in *E. coli* BL21(DE3) (0.3 mM IPTG, 25°C, 20 h), and the lysates were added to the PA2072‐bound GFP‐Trap beads and incubated for 2 h at 4°C with gentle rotation. The beads were then washed and eluted as above. Coprecipitation samples were analyzed using anti‐GFP (Abcam, United Kingdom, diluted 1:10,000) and anti‐mCherry antibodies (Proteintech, United States, diluted 1:3,000), respectively.

### 2.14. *β*‐Galactosidase (*β*‐GAL) Activity Analysis

*β*‐GAL activity was measured in transformants grown for 24 h in LB medium (containing 100 *μ*g/mL AMP, 50 *μ*g/mL KAN, and 0.05 mM IPTG) at 37°C. Cells were pelleted (12,000 × g, 4°C, 2 min) and analyzed using a *β*‐GAL Assay Kit (Solarbio, Beijing, China).

### 2.15. Statistical Analysis

Data represent the mean ± standard error of the mean (SEM) from three independent biological replicates. Statistical analyses were performed using GraphPad Prism (Version 6.0). Differences between groups were evaluated using one‐way analysis of variance (ANOVA), two‐way ANOVA as appropriate, with statistical significance defined as *p* < 0.05.

## 3. Results

### 3.1. PA2072 Is Involved in Diversified Biological Functions

**Figure 1:**
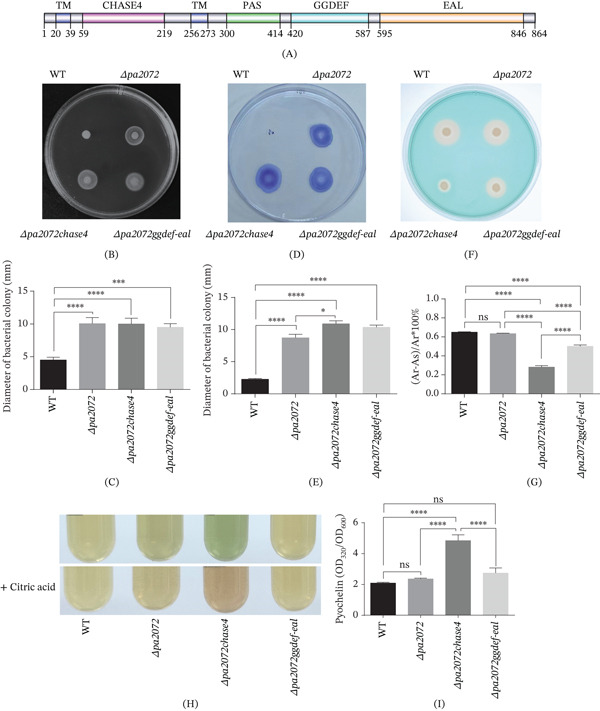
Phenotypic analysis of *pa2072* mutants. (A) Schematic depiction of PA2072 molecular architecture. Domains were predicted using the NCBI Conserved Domain Database (https://www.ncbi.nlm.nih.gov/Structure/cdd/wrpsb.cgi), and transmembrane regions were predicted using the TMHMM server (http://www.cbs.dtu.dk/services/TMHMM/). Amino acid boundaries for each domain were indicated. (B, D, F) Representative agar plates showing the (B) swimming motility, (D) twitching motility, and (F) siderophore production of wild‐type PAO1 and the *pa2072* mutant strains (*Δpa2072*, *Δpa2072chase4*, and *Δpa2072ggdef-eal*, for full length, CHASE4, and GGDEF–EAL domain deletions, respectively). (C) Quantitative representation of swimming motility, showing colony diameters (millimeters) in wild‐type PAO1 and the *pa2072* mutant strains (*n* = 3). (E) Quantitative representation of twitching motility, showing colony diameters (millimeters) in wild‐type PAO1 and *the pa2072* mutant strains (*n* = 4). (G) Quantifying siderophore production by mixing CAS solution with the supernatant, followed by spectrophotometric measurements at OD_630_ (*n* = 3). (H) Representative picture of pyochelin extracted from wild‐type PAO1 and the three *pa2072* mutants. (I) Spectrophotometric quantification of pyochelin by measuring the ethyl acetate–extracted supernatant at OD_320_ and normalized to cell density (OD_600_) (*n* = 3). Data represent mean ± standard error of the mean (SEM), and statistical significance was determined by one‐way ANOVA followed by Tukey′s post hoc test for multiple comparisons:  ^∗^ *p* < 0.05,  ^∗∗∗^ *p* < 0.001, and  ^∗∗∗∗^ *p* < 0.0001; ns, no significance.

A total of 42 c‐di‐GMP metabolizing proteins are encoded in the *P. aeruginosa* PAO1 genome [19], including PA2072, which is composed of 864 amino acid residues, with its N‐terminal CHASE4 domain flanked by two transmembrane segments, followed by an intracellular PAS domain and a C‐terminal GGDEF–EAL dual‐domain module (Figure 1A). As PA2072 contains a complicated molecular architecture, we constructed three deletion mutants: the full‐length deletion mutant of *Δpa2072* (1–864 aa), the CHASE4 domain deletion mutant of *Δpa2072chase4* (59–219 aa), and the GGDEF–EAL domain deletion mutant of *Δpa2072ggdef-eal* (420–864 aa) (Figure 1, also see Materials and Methods).

Phenotypic analysis was performed. As shown in Figure 1B–I, compared with wild‐type PAO1, swimming and twitching motility were increased in all three mutant strains, with no significant differences among the mutants (Figure 1B,E). The CAS agar plate assay is a method used to analyze siderophore production [20], in which the grabbing of Fe^3+^ by the bacterial siderophores frees the CAS dye from the Fe^3+^–dye complex, leading to a color change from blue to orange. In our analysis, *P. aeruginosa* PAO1 and all three *pa2072* mutants exhibited the ability to produce siderophores (Figure 1F). The three PA2072 mutant strains seemed to produce different amounts of siderophores, with the CHASE4 domain deletion mutant *Δpa2072chase4* producing the least siderophores (Figure 1F,G). We also tested the ability of the different strains to produce pyochelin, a green‐colored siderophore [21]. Interestingly, compared with wild‐type PAO1, full‐length PA2072 deletion *Δpa2072* and C‐terminus GGDEF–EAL deletion *Δpa2072ggdef-eal* produced roughly similar amounts of pyochelin, whereas that in the *Δpa2072chase4* increased drastically (Figure 1H,I). These observations imply that, as a protein with a complicated structure, the PA2072 individual domains may be involved in profound but sometimes different functions in bacterial physiology.

### 3.2. PA2072 Displays DGC Activity

**Figure 2:**
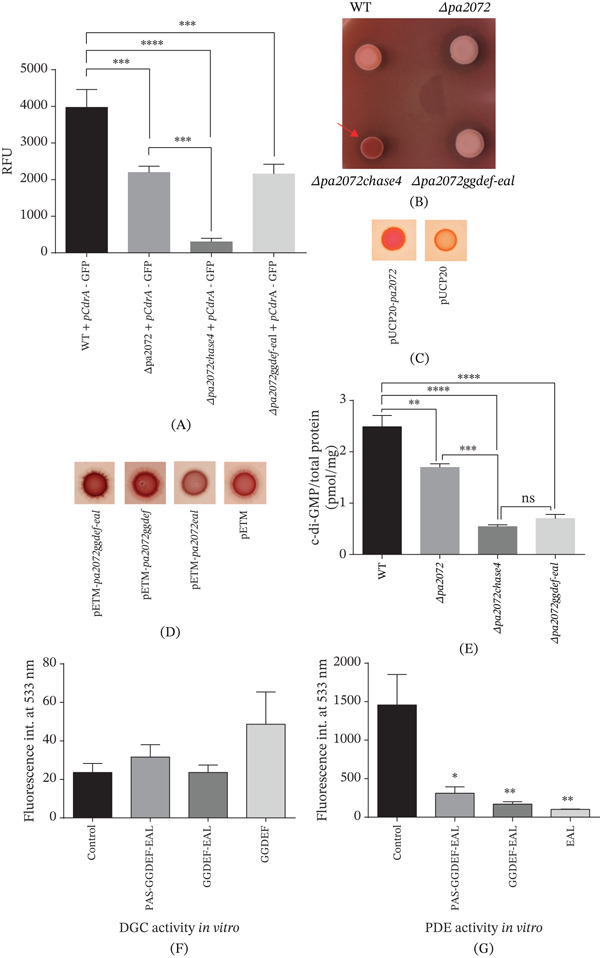
PA2072 contains DGC activity. (A) Intracellular c‐di‐GMP quantification using p*CdrA*‐GFP reporter plasmid. GFP fluorescence was measured in stationary‐phase samples. Values represented relative fluorescence units (RFUs), which were normalized to cell density (OD_600_). Data were from three independent experiments; statistical significance was determined by one‐way ANOVA followed by Tukey′s post hoc test for multiple comparisons. (B) Congo Red (CR) plate assays. *P. aeruginosa* wild‐type PAO1 and the mutant strains (*Δpa2072*, *Δpa2072chase4*, and *Δpa2072ggdef-eal*) were grown on LB agar with 40 *μ*g/mL CR and 20 *μ*g/mL Coomassie Brilliant Blue G‐250. (C, D) CR plate assays. *E. coli* BL21(DE3) strains containing the recombinant plasmid of the full‐length PA2072 cloned in a (C) pUCP20 vector or the PA2072 GGDEF–EAL, GGDEF, and EAL domains cloned separately in a (D) pETM vector, cultured on M9 agar supplemented with 20 *μ*g/mL CR and 0.05 mM IPTG. (E) Determination of the intracellular c‐di‐GMP level using LC‐MS measurement. Values were normalized to total cellular protein content. (F, G) Thiazole orange–derived fluorometric assay to analyze DGC and PDE activities in vitro. Recombinant proteins of the individual domains of PA2072 were purified and adjusted to a concentration of 15 *μ*M. The concentrations of c‐di‐GMP were monitored by measurements of fluorescent thiazole orange at the wavelength of 533 nm. Data representing three independent biological replicates was determined by one‐way ANOVA:  ^∗^ *p* < 0.05,  ^∗∗^ *p* < 0.01,  ^∗∗∗^ *p* < 0.001, and  ^∗∗∗∗^ *p* < 0.0001; ns, no significance.

The biological functions of c‐di‐GMP proteins are related to their enzymatic activity. As shown in Figure 2A, the p*CdrA*‐GFP fluorescence reporter assay [17] was employed to gauge the in vivo c‐di‐GMP level, and the mutant strains *Δpa2072*, *Δpa2072chase4*, and *Δpa2072ggdef-eal* showed lower c‐di‐GMP concentrations, indicating the overall DGC activity of PA2072. The CR plate assay is a method that links cellular c‐di‐GMP level with colony coloration [22]. In Figure 2B, the full‐length PA2072 deletion mutant of *Δpa2072* and the GGDEF–EAL domain deletion mutant of *Δpa2072ggdef-eal* both resulted in paler coloration, representing reduced c‐di‐GMP formation. Noticeably, *Δpa2072chase4* exhibited a smaller colony with redder coloration (Figure 2B, arrow), though its cellular c‐di‐GMP level was suggested to decrease (Figure 2A). In a recombinant construct expression assay, the full‐length PA2072 cloned in the pUCP20 vector (Figure 2C) and the enzymatic domains cloned separately in a pETM vector (Figure 2D) were transformed into *E. coli* BL21(DE3) host cells. Compared with the vector controls, the full‐length PA2072, GGDEF–EAL, and GGDEF domains displayed redder colors on the CR plates (Figure 2C,D), whereas the EAL domain displayed a paler color (Figure 2D). These observations were further confirmed by the in vitro assays: In LC‐MS measurements (Figure 2E), the full‐length PA2072 and the domain‐specific deletion mutants showed a much lower cellular c‐di‐GMP concentration compared with that of the wild‐type PAO1 sample. Additionally, direct enzymatic reactions indicated that both DGC and PDE activities could be detected in tubes (Figure 2F,G), implying the complexity of domain functions in the c‐di‐GMP metabolizing proteins. In total, PA2072 showed DGC activity with its GGDEF–EAL dual‐domain structure, in accordance with those reported by Cai et al. [23].

### 3.3. Transcriptional Analysis

**Figure 3:**
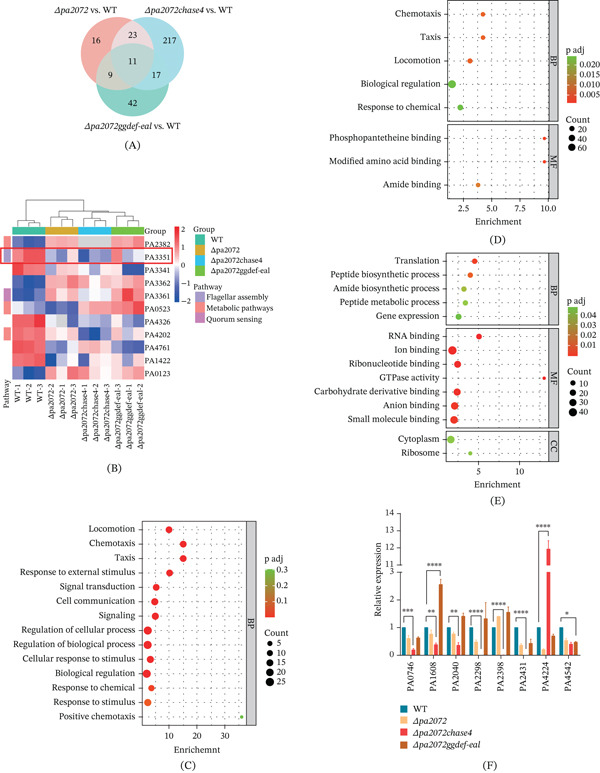
Transcriptomic analysis of PA2072 mutants. (A) Venn diagram depicting differentially expressed genes (DEGs) in different comparisons (*Δpa2072* vs. WT, *Δpa2072chase4* vs. WT, and *Δpa2072ggdef-eal* vs. WT) and their intersections. DEGs were defined with the thresholds of |log2 (*f* *o* *l* *d* *c* *h* *a* *n* *g* *e*)| ≥ 1 and *p* adj ≤ 0.05. (B) Heatmap displaying expression abundance of all identified DEGs in wild‐type PAO1 and the three PA2072 mutant strains. The red box is marked for *pa3351* (*flgM*), a gene related to swimming and twitching motility in PA2072 mutant strains. (C–E) Gene Ontology (GO) enrichment analysis of DEGs. The top significantly enriched terms (*p* adj ≤ 0.05) in biological process (BP), cellular component (CC), and molecular function (MF) were shown, respectively: (C) *Δpa2072* versus WT, (D) *Δpa2072chase4* versus WT, and (E) *Δpa2072ggdef-eal* versus WT. (F) qRT‐PCR analysis of transcriptional levels for selected DEGs using SYBR Green, which were normalized to *rpoD* in wild‐type PAO1. The relative expression level in each mutant was calculated using the 2^−*ΔΔ*CT^ method. Data represented mean ± SEM from three independent biological replicates. Statistical significance was determined by two‐way ANOVA with Tukey′s post hoc test for multiple comparisons:  ^∗^ *p* < 0.05,  ^∗∗^ *p* < 0.01,  ^∗∗∗^ *p* < 0.001, and  ^∗∗∗∗^ *p* < 0.0001.

We investigated the transcriptional differences between the WT PAO1 and the three PA2072 mutants. A supporting RNA‐seq file including the detailed statistical data was provided (Supporting Information 6: File S1). Differentially expressed genes (DEGs) were identified using DESeq2 with stringent thresholds (|log2 (fold change)| ≥ 1 and *p* adj ≤ 0.05). Venn analysis identified 11 common DEGs in the three groups, with 16 DEGs in *Δpa2072* versus WT, 41 in *Δpa2072ggdef-eal* versus WT, and 217 in *Δpa2072chase4* versus WT (Figure 3A).

The heatmap clustering of the 11 common DEGs was mainly enriched in pathways critical for bacterial adaptation, such as chemotaxis, nitrogen metabolism, and quorum sensing (Figure 3B). Noticeably, the negative regulator of flagellar motility FlgM (PA3351, Figure 3B box) was downregulated in all mutant strains, suggesting enhanced motility, which was consistent with the phenotypic observations of motility (Figure 1B–E).

GO enrichment analysis was then performed. As shown in Figure 3C, most of the DEGs (*Δpa2072* vs. WT) were enriched for chemotaxis, motility, sensing of external stimuli, locomotion, and signal transduction (Figure 3C and Table 1). The DEGs of *Δpa2072chase4* versus WT were mainly enriched for chemotaxis, motility, bioregulation, locomotion, siderophore synthesis and metabolism, and amino acid binding (Figure 3D and Table 2). Interestingly, two genes, *pa2398* (pyoverdine receptor FpvA, log_2_FC = −8.4) [24] and *pa2397* (pyoverdine synthase PvdE, log_2_FC = −8.1) [25], showed obvious downregulation (Table 2, bold), which is consistent with the CAS results of low‐level siderophore production in this strain (Figure 1F,G). Additionally, the most significantly upregulated gene, *pa4224* (log_2_FC = 7.9), encodes the pyochelin biosynthesis protein PchG [21] (Table 2, bold). This result was consistent with the *Δpa2072chase4* pyochelin phenotype assay (Figure 1H,I). As for *Δpa2072ggdef-eal* versus WT, the DEGs were enriched in the molecular functions of GTPase activity, RNA binding, ion binding, binding of carbohydrate derivatives and small molecules, and the biological processes of translation, peptide biosynthesis and metabolic processes, and amide biosynthetic processes (Figure 3E, Table 3).

**Table 1:** The 20 most significantly upregulated and downregulated genes in *Δpa2072* versus WT PAO1.

Gene_name	Gene_ID	Product	Log_2_FC	FDR‐adjusted *p* value
Upregulated (fold change ≥ 1.3 plus **p** ≤ 0.05)				
*pa0528*	*pa0528*	Transcriptional regulator	2.366673	0.038466418
*pa4350*	*pa4350*	Hypothetical protein	2.220389	1.51e − 09
*lecB*	*pa3361*	Fucose‐binding lectin PA‐IIL	2.154173	1.25e − 05
*pa5474*	*pa5474*	Metalloprotease	1.933406	5.44e − 09
*lldA*	*pa2382*	L‐Lactate dehydrogenase	1.844627	0.000190535
*pa4351*	*pa4351*	Acyltransferase	1.818431	1.10e − 09
*gltB*	*pa5036*	Glutamate synthase (NADPH) large chain	1.781202	1.26e − 09
*pa2503*	*pa2503*	Hypothetical protein	1.700478	0.01935254
*fpvA*	*pa2938*	Ferripyoverdine receptor	1.399191	0.002172258
*nusA*	*pa4745*	Transcription elongation factor NusA	1.376532	0.000682334
Downregulated (fold change ≤ −2 plus **p** ≤ 0.05)				
*gbuR*	*pa1422*		−2.82488	0.011595716
*motA*	*pa4954*	Flagellar motor protein MotA	−2.6774	0.015117147
*pa1679*	*pa1679*	Hypothetical protein	−2.64303	7.50e − 05
*pa1608*	*pa1608*	Chemotaxis transducer	−2.52887	4.50e − 05
*pa2867*	*pa2867*	Chemotaxis transducer	−2.51843	5.34e − 18
*pa1095*	*pa1095*	B‐type flagellar protein FliS	−2.3578	2.62e − 13
*pa2920*	*pa2920*	Chemotaxis transducer	−2.35028	4.67e − 07
*bkdA2*	*pa2248*	2‐Oxoisovalerate dehydrogenase subunit beta	−2.17804	0.004396402
*dnaK*	*pa4761*	Molecular chaperone DnaK	−2.16786	7.97e − 07
*bdlA*	*pa1423*	Biofilm dispersion protein	−2.09193	0.020357735

**Table 2:** The 20 most significantly upregulated and downregulated genes in *Δpa2072chase4* versus WT PAO1.

Gene_name	Gene_ID	Product	Log_2_FC	FDR‐adjusted *p* value
Upregulated (fold change ≥ 5.31 plus *p* ≤ 0.05)				
***pchG***	***pa4224***	Pyochelin biosynthesis protein PchG	7.908663	4.73e − 10
*pchB*	*pa4230*	Isochorismate pyruvate lyase	6.428539	0.003132787
*aprE*	*pa1247*	Alkaline protease secretion protein AprE	6.267109	3.34e − 05
*pchE*	*pa4226*	Dihydroaeruginoic acid synthetase	5.86027	1.69e − 08
*pa2540*	*pa2540*	Hypothetical protein	5.798981	6.19e − 20
*pa4222*	*pa4222*	ABC transporter ATP‐binding protein	5.713414	0.010000427
*pa3205*	*pa3205*	Periplasmic protein CpxP/Spy	5.570452	1.50e − 14
*pa2691*	*pa2691*	Hypothetical protein	5.551779	0.002126428
*pchA*	*pa4231*	Salicylate biosynthesis isochorismate synthase	5.38404	3.97e − 08
*pchF*	*pa4225*	Pyochelin synthetase	5.314062	3.18e − 09
Downregulated (fold change ≤ −8.19 plus *p* ≤ 0.05)				
*pa2458*	*pa2458*	Hypothetical protein	−9.85918	1.30e − 13
*bkdB*	*pa2249*	Branched‐chain alpha‐keto acid dehydrogenase complex lipoamide acyltransferase	−9.14458	1.67e − 11
*safC*	*pa2462*	Outer membrane fimbrial usher protein	−8.75754	1.82e − 10
*pa2491*	*pa2491*	Oxidoreductase	−8.73553	2.29e − 10
*pa2527*	*pa2527*	Resistance‐nodulation‐cell division (RND) efflux transporter	−8.72586	3.03e − 10
*pa2483*	*pa2483*	Hypothetical protein	−8.58542	5.58e − 10
***fpvA***	***pa2398***	Ferripyoverdine receptor	−8.44444	1.54e − 09
*pa2402*	*pa2402*	Peptide synthase	−8.39909	1.54e − 09
*pa2500*	*pa2500*	Major facilitator superfamily transporter	−8.32881	3.81e − 09
***pvdE***	***pa2397***	Pyoverdine biosynthesis protein PvdE	−8.19632	5.50e − 09

*Note:* Bold format indicate DEGs associated with siderophore biosynthesis (including pyochelin and pyoverdine). See text for details.

**Table 3:** The 20 most significantly upregulated and downregulated genes in *Δpa2072ggdef-eal* versus WT PAO1.

Gene_name	Gene_ID	Product	Log_2_FC	FDR‐adjusted *p* value
Upregulated (fold change ≥ 2.35 plus **p** ≤ 0.05)				
*pa0122*	*pa0122*	Hypothetical protein	3.159762	1.24e − 06
*rplK*	*pa4274*	50S ribosomal protein L11	3.157098	0.00840205
*lecB*	*pa3361*	Fucose‐binding lectin PA‐IIL	3.146104	2.11e − 09
*nirQ*	*pa0520*	Denitrification regulatory protein NirQ	3.042906	0.038466738
*norB*	*pa0524*	Nitric oxide reductase subunit B	2.893411	9.28e − 13
*pepA*	*pa3831*	Leucyl aminopeptidase	2.865968	0.005351687
*pa3362*	*pa3362*	Transporter protein AmiS	2.603042	3.50e − 07
*nirS*	*pa0519*	Nitrite reductase	2.450268	0.000209684
*pa1127*	*pa1127*	Oxidoreductase	2.410553	0.030379555
*pa1656*	*pa1656*	Type VI secretion system protein VasJ	2.350702	0.001247279
Downregulated (fold change ≤ −2.31 plus **p** ≤ 0.05)				
*pa4326*	*pa4326*	Hypothetical protein	−4.31374	1.41e − 05
*pa0741*	*pa0741*	Hypothetical protein	−2.8069	0.007775672
*pa1922*	*pa1922*	TonB‐dependent receptor	−2.79286	0.010292234
*pa3401*	*pa3401*	ABC‐2 type transport system permease protein	−2.78708	0.009000089
*bfiS*	*pa4197*	Protein BfiS	−2.75912	5.66e − 10
*pa1897*	*pa1897*	Hypothetical protein	−2.73073	0.009000089
*dnaK*	*pa4761*	Molecular chaperone DnaK	−2.59528	3.12e − 09
*copS*	*pa2810*	Two‐component sensor CopS	−2.50995	6.14e − 06
*pa4513*	*pa4513*	Oxidoreductase	−2.31843	9.59e − 06
*htpG*	*pa1596*	Chaperone protein HtpG	−2.31024	0.011391663

Real‐time PCR was performed using *rpoD* as the internal reference (Figure 3F). The expression levels of the selected pyochelin biosynthesis‐related gene PA4224, pyoverdine transporter PA2398, oxidoreductase gene PA2298, acyl‐coenzyme dehydrogenase gene PA0746, glutamate synthase gene PA2040, ATP‐dependent Clp protease PA4542, and putative genes PA2431 and PA1608 were analyzed, and the pattern was highly consistent with the RNA‐seq results (Figure 3F).

### 3.4. BTH Analysis

**Figure 4:**
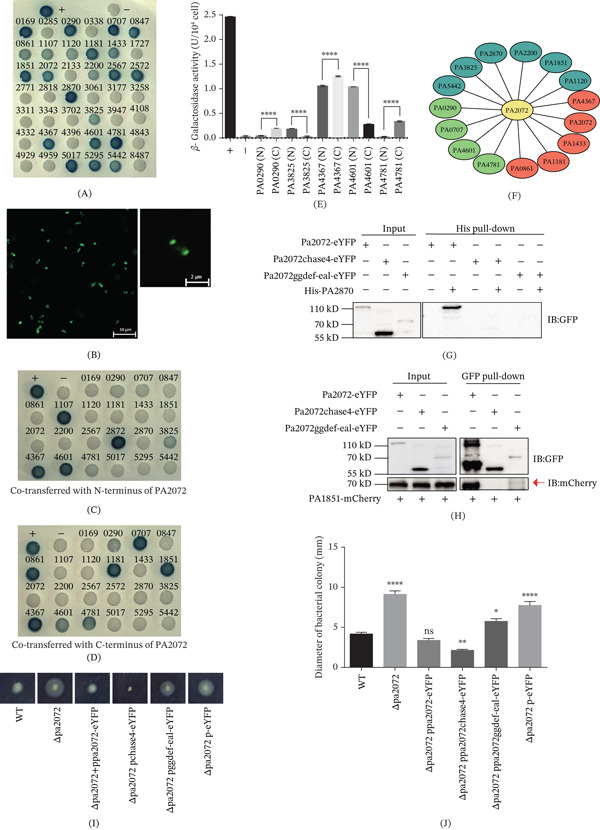
In vitro analysis of protein–protein interactions (PPIs) of PA2072 with other c‐di‐GMP metabolizing proteins in *P. aeruginosa*. (A) Bacterial two‐hybrid (BTH) screening PPIs between PA2072 and 42 c‐di‐GMP metabolizing proteins. PPIs were tested using *E. coli* BTH101 cells grown on LB agar with X‐gal. Blue coloration indicated positive interactions. Each pair was tested reciprocally (pUT18C and pKT25 fusions). +, positive control; −, negative control. (B) Cellular localization of PA2072‐eYFP in the *Δpa2072* strain. A fluorescent image was acquired by confocal microscopy (63x oil‐immersion objective, 4x digital zoom). Scale bars: 10 *μ*m (left) and 2 *μ*m (right). (C, D) BTH analysis of the *pa2072* (C) N‐terminal CHASE4 domain or (D) C‐terminal GGDEF–EAL domain with the interactive partners. (E) Quantitative analyses to measure the interaction strength by *β*‐galactosidase activity levels in *E. coli* BTH101 cells. (F) PPI networking of PA2072 was generated using Cytoscape (Version 3.9.1). Red nodes representing PA2072 interaction with these proteins from four independent experiments, blue nodes representing three, and green representing two (Supporting Information 4: Table S3). (G) Ni‐NTA pull‐down of His‐PA2870 (59 kDa) with PA2072‐eYFP (121 kDa), PA2072chase4‐eYFP (57 kDa), or PA2072ggdef‐eal‐eYFP (75 kDa). Input and His pull‐down fractions were immunoblotted with anti‐GFP monoclonal antibody (diluted 1:10,000). “+” and “−” denote the presence and absence of the indicated component, respectively. (H) GFP‐Trap pull‐down of PA1851‐mCherry (71 kDa) with the indicated PA2072‐eYFP, PA2072chase4‐eYFP, or PA2072ggdef‐eal‐eYFP. Input and GFP pull‐down fractions were blotted using anti‐GFP or anti‐mCherry monoclonal antibody (diluted 1:3000). (I) Swimming motility of wild‐type PAO1, *Δpa2072* and the *pa2072* complementation strains (*Δpa2072*p*pa2072-eYFP*, *Δpa2072*p*pa2072chase4-eYFP*, and *Δpa2072*p*pa2072ggdef-eal-eYFP*), and *Δpa2072*p*-eYFP* was used as the negative control. (J) Quantitative representation of swimming motility, showing colony diameters (millimeters) in wild‐type PAO1 and the *pa2072* complementation strains. Data represent mean ± SEM; one‐way ANOVA:  ^∗^ *p* < 0.05,  ^∗∗^ *p* < 0.01, and  ^∗∗∗∗^ *p* < 0.0001; ns, no significance.

c‐di‐GMP metabolizing protein functions are closely related to PPIs, and the BTH assay (Euromedex, France) is a straightforward and efficient method [21]. Three pUT18C constructs were constructed: full‐length PA2072, N‐terminus (1–280 aa, including the CHASE4 domain and two transmembrane regions), and C‐terminus (420–864 aa, including GGDEF and EAL domains). Among the 42 c‐di‐GMP metabolizing proteins cloned into the pKT25 or pKNT25 vectors [18], full‐length PA2072 interacted with many of them (Figure 4A, Supporting Information 4: Table S3). Additionally, in an orthogonal validation where PA2072 was cloned in a T25 vector and the rest were cloned into a T18 vector, five interacting pairs were verified (Figure 4F). Six interacting pairs appeared both in our study and Zhan et al.′s [26]; another four interactions were repeated and proved further in our lab (Figure 4F). From the *pa2072* gene organization analysis (Figure 1A), it was clear that PA2072 is a membrane‐anchored protein. We performed eYFP labeling analysis and found that PA2072 was bipolarly localized (Figure 4B). We then split PA2072 into an N‐terminal transmembrane sensor region and a C‐terminal catalytic region and applied BTH analysis. Intriguingly, for the 22 possible interacting partners, seven paired with the N‐terminal CHASE4 region, whereas two of them, PA1107 and PA2572, seemed to contact this region alone (Figure 4C, Supporting Information 5: Table S4), nine interacted with the C‐terminal GGDEF–EAL region of PA2072, and four of them, PA0707, PA0861, PA1181, and PA1851, seemed to interact with this segment exclusively (Figure 4D, Supporting Information 5: Table S4). Five genes, PA0290, PA3825, PA4367, PA4601, and PA4781, interacted with both the segments. We also performed a simple assay for LacZ enzymatic activity. As shown in Figure 4E, the interacting strength of the five genes with the PA2072 N‐ and C‐terminal regions varied significantly. In general, the c‐di‐GMP metabolizing genes most likely to interact with PA2072 were summarized and shown as a Cytoscape image in Figure 4F.

In order to consolidate the BTH results, in vitro biochemical assays were applied. The proteins of PA2072, PA2870, and PA1851 were cloned into prokaryotic recombinant expression systems, respectively. As shown in Figure 4G, eYFP‐tagged full‐length PA2072 was coprecipitated with His‐tagged PA2870, whereas PA2072 N‐ or C‐terminal fragments displayed no signal. Protein PA1851 contains several transmembrane segments and was poorly expressed in His‐tag vectors (data not shown); this protein was then tagged with mCherry. Using the GFP‐Trap magnetic beads, PA1851‐mCherry was pulled down by the full‐length and C‐terminal GGDEF–EAL domain of PA2072 (Figure 4H, arrow), but showed no interactions with PA2072 N‐terminal CHASE4 domain (1–280 aa). These results are in agreement with our BTH assays but different from what Zhan et al. reported. One possible reason is that the segments are cloned differently: We focused on the N‐terminal signal‐sensing CHASE4 and the C‐terminal catalytic GGDEF–EAL domain, with no PA2072 PAS‐PAC domain included in our BTH design or in the pull‐down constructs.

## 4. Discussion

c‐di‐GMP is a novel bacterial secondary messenger that is involved in diverse physiological processes. The c‐di‐GMP metabolizing proteins display genetic redundancy; for example, multiple DGCs and PDEs are encoded in one genome. Meanwhile, these proteins show precise signaling specificity, where extensive efforts have been made to understand the underlying mechanisms, among which PPIs are of great significance [9].

Researchers have recently suggested that there are two types of c‐di‐GMP metabolizing protein interactions: the “simple local signaling” and the “complex local signaling” manner [9]. Additionally, it has long been noticed that c‐di‐GMP proteins adopt complicated molecular organization: The N‐terminal sensory domains, such as CHASE, PAS, and GAF, are usually concatenated to the C‐terminal catalytic GGDEF and/or EAL domains. However, the exact functions of the individual domains and their roles in these interactions are largely unknown.

In the current study, we generated full‐length and domain deletions of PA2072, a c‐di‐GMP metabolizing protein from *P. aeruginosa* PAO1. As shown in Figure 2, intracellular measurements, CR colonies, and recombinant expression assays illustrated that the dual PA2072 GGDEF–EAL domain mainly acted as a DGC, though PDE activity could be detected in vitro. Accordingly, swimming and twitching motility were enhanced in the deletion mutants (Figure 1B,D), probably because of the reduced c‐di‐GMP synthetic activity and decreased intracellular c‐di‐GMP levels.

The observation of individual strains producing siderophores differentially among the mutants (Figure 1F,H) may be linked to the expression profile analysis (Table 2). The decreased total siderophore production in the CHASE4 domain deletion strain (Figure 1F,G) might be associated with the decreased expression of PA2397 (pyoverdine synthase PvdE) and PA2398 (ferripyoverdine receptor) (Table 2). A recent publication [26] showed that PA2072 CHASE4 is involved in iron sensing. Our results might further link the deletion of this iron‐sensing domain and the expression regulation of iron‐scavenging pyoverdine synthetic genes. Clearly, more work is needed in the future.

*P. aeruginosa* PAO1 encodes abundant c‐di‐GMP metabolizing proteins. These c‐di‐GMP proteins interact with each other, forming a complicated network [9], whose role in physiology is still difficult to predict. In our BTH analysis, PA2072 was found to interact with a number of PAO1 c‐di‐GMP metabolizing proteins, including PA2072 itself. Compared with a recent publication [26], we identified more interacting pairs, which were further confirmed by MacConkey medium analysis (Supporting Information 1: Figure S1). The detailed information is summarized in Supporting Information 4: Table S3 and presented in Figure 4F. Importantly, we separated PA2072 into the N‐terminal CHASE4 and the C‐terminal GGDEF–EAL regions and found that the interacting behavior for each domain was unique and different from that of the full‐length PA2072, which was validated further by our in vitro biochemical assays (Figure 4G,H).

In the current stage, it is challenging to link the c‐di‐GMP metabolizing molecules′ interactions to individual bacterial phenotypes. In our complementary experiment, it could be observed that the full‐length PA2072 compensated for the swimming motility of *Δpa2072* roughly to wild‐type level (Figure 4I,J). Differently, the construct of the CHASE4 domain brings *Δpa2072* a significantly decreased swimming motility (Figure 4I,J). The CHASE4 domain does not contain catalytic activity, but highly involved in signal sensing: Zhan et al. reported its function of iron sensing [26], our analysis displayed its pivotal roles in extracellular ATP sensing [27], and very recently, Angeli et al. published their work, showing the CHASE4 domains′ versatility for sensing of different environmental cues, including heme and copper [28]. It is reasonable to deduce that diversified environmental stimuli sensed by the CHASE4 domain may facilitate downstream signal transduction and integration of the c‐di‐GMP networking, mainly via the domain‐specific interactions and cross‐talk, which could be more complex than previously hypothesized.

It has long been noted that in c‐di‐GMP signaling proteins, ligand binding, domain dimerization and rearrangement, and changes in enzymatic activity are of significance [29]. Multiple PPIs have been described in this field [16]. Full‐length protein interactions are usually recognized, whereas individual domain functions and their roles in interactions are missing. In the current study, we found that the PA2072 CHASE4 and GGDEF–EAL domains are related to different physiological functions of siderophore production and motility. Additionally, they display different c‐di‐GMP metabolizing PPI patterns. These observations are novel and likely to be biologically meaningful, helping deepen our understanding of this rapidly developing field.

## 5. Conclusion

In the current study, we found that the dual c‐di‐GMP metabolizing protein PA2072 from *P. aeruginosa* PAO1 acted as a DGC and was involved in complicated physiological processes. Intriguingly, as a multidomain protein, the PA2072 N‐terminal CHASE4 domain was involved in siderophore production, and its C‐terminal catalytic GGDEF–EAL domain was linked to motility. Functional differentiation may be associated with the different PPI patterns. Further analysis is required in the near future.

## Author Contributions

**Xiaojing Gao**: investigation, methodology, data curation, formal analysis, visualization, and writing—original draft; **Yan Zhang**: experiments and formal analysis of enzymatic activity; **Lingna Guo**: investigation in molecular part; **Huimin Zhang**: BTH vector construction and sequencing analysis; **Jianwen Sun**: investigation in molecular part; **Jianfang Sun**: writing—review and editing; **Weidong Huang**: funding acquisition, conceptualization, methodology, experimental design, data analysis, resources, writing—original draft, and writing—review and editing. **Xiaojing Gao** and **Yan Zhang** contributed equally to this work.

## Funding

This work was supported by the Natural Science Foundation of Ningxia Hui Autonomous Region (2024AAC03266) and the Ningxia Key Research and Development Project (2022BEG03151).

## Conflicts of Interest

The authors declare no conflicts of interest.

## Supporting Information

Additional supporting information can be found online in the Supporting Information section.

## Supporting information


**Supporting Information 1** Figure S1: The interaction between PA2072 and other c‐di‐GMP metabolizing proteins in *P. aeruginosa* PAO1, as analyzed in MacConkey medium for this paper.

**Supporting Information 2** Table S1: A list of the bacterial strains and plasmids used in this study.

**Supporting Information 3** Table S2: A list of primer sequences employed in this study.

**Supporting Information 4** Table S3: The protein–protein interaction data between PA2072 and 42 c‐di‐GMP metabolizing proteins in *P. aeruginosa* in this study.

**Supporting Information 5** Table S4: Summary of the molecular architecture of the 22 c‐di‐GMP metabolizing proteins in *P. aeruginos*a PAO1 that exhibited interactions with PA2072 in this study.

**Supporting Information 6** File S1: The biological replicate number, sequencing depth, PCA plots, quality metrics, dispersion, and read mapping statistics in this study.

## Data Availability

The datasets generated/analyzed during the current study are available in the Figshare repository (10.6084/m9.figshare.32739432).
