# Supplementary material for: Diverse Biological Functions and Domain‐Specific Interactions of the Diguanylate Cyclase PA2072 in Pseudomonas aeruginosa PAO1
Source: Int J Microbiol. 2026 Jul 18;2026:7292171. doi: 10.1155/ijm/7292171 (PMC13380032; doi:10.1155/ijm/7292171)
Supplement: Supplementary file 1 — Supporting Information 1 Figure S1: The interaction between PA2072 and other c‐di‐GMP metabolizing proteins in P. aeruginosa PAO1, as analyzed in MacConkey medium for this paper. [file IJM-2026-7292171-s006.docx]

PA2072 interacts with other c-di-GMP metabolizing proteins in *P. aeruginosa* PAO1, analyzed in MacConkey medium


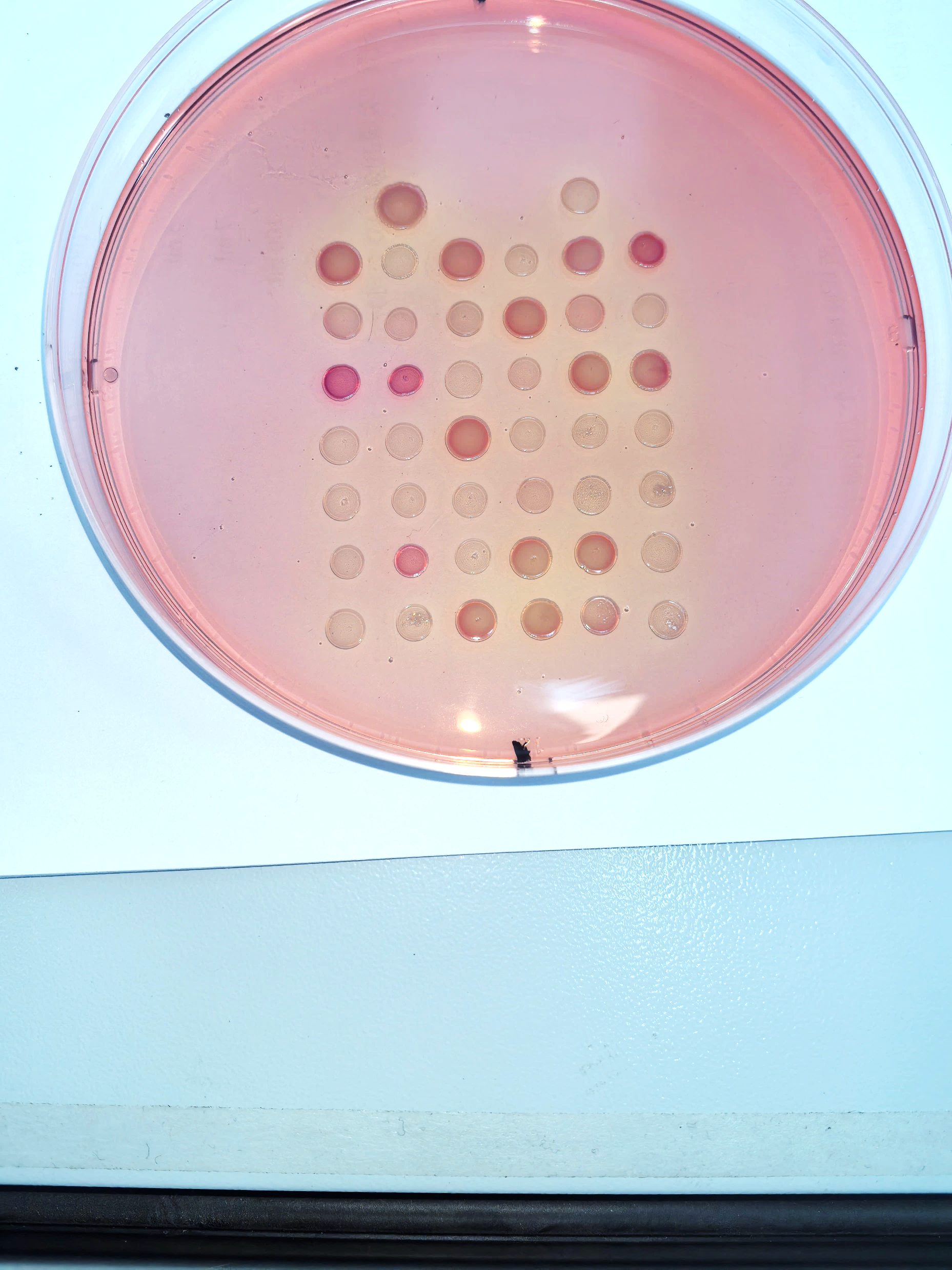


+

-

0169 0285 0290 0338 0707 0847

0861 1107 1120 1181 1433 1727

1851 2072 2133 2200 2567 2572

2771 2818 2870 3061 3177 3258

3311 3343 3702 3825 3947 4108

4332 4367 4396 4601 4781 4843

4929 4959 5017 5295 5442 5487

Figure S1. Bacterial-two-hybrid (BTH) analysis of PA2072 with other c-di-GMP metabolizing proteins in *P. aeruginosa* PAO1. Strains were cultured on MacConkey agar with 0.5 mM IPTG, 100 μg/mL ampicillin, 50 μg/mL kanamycin and 0.2% lactose, at 30 ºC for 24 h. ‘+’ and ‘−’ represent positive and negative controls (Euromedex BACTH system, France).
