# Supplementary material for: Diverse Biological Functions and Domain‐Specific Interactions of the Diguanylate Cyclase PA2072 in Pseudomonas aeruginosa PAO1
Source: Int J Microbiol. 2026 Jul 18;2026:7292171. doi: 10.1155/ijm/7292171 (PMC13380032; doi:10.1155/ijm/7292171)
Supplement: Supplementary file 6 — Supporting Information 6 File S1: The biological replicate number, sequencing depth, PCA plots, quality metrics, dispersion, and read mapping statistics in this study. [file IJM-2026-7292171-s005.docx]

Supplementary information for RNA-Seq Analysis

Biological Replicate Number: In current study, RNA-seq analysis of 4 experimental groups, with 3 independent biological replicates in each group, was applied.

Sequencing Depth: The average sequencing depth was approximately 40 million paired-end reads per sample.

PCA Plots: The PCA plots showing the clustering of biological replicates have been provided as follows:


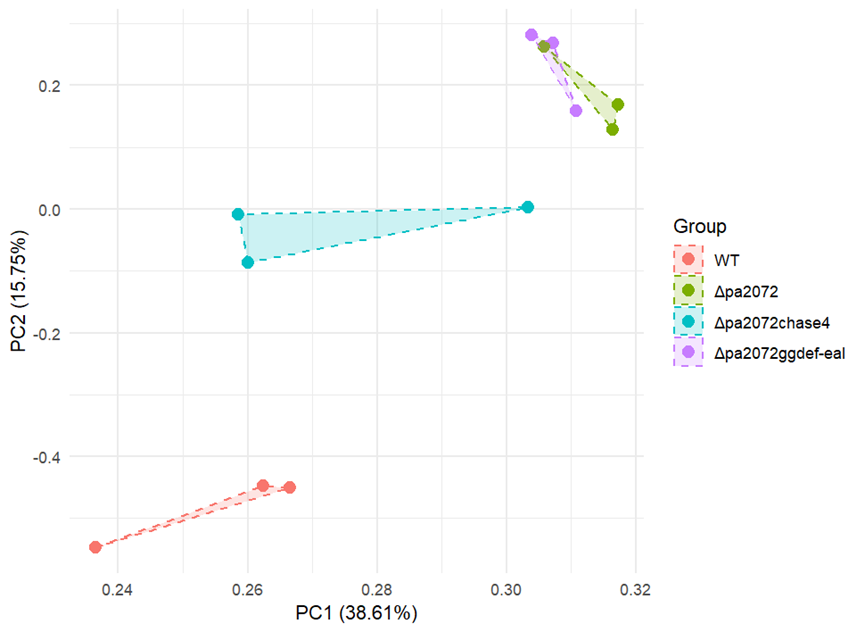
PCA Analysis of Different Groups

Seq Figure 1. Principal component analysis (PCA) of *P. aeruginosa* wild-type PAO1 and the mutant strains (*Δpa2072*, *Δpa2072chase4*, *Δpa2072ggdef-eal*). The figure shows a clear separation between the wild-type and the three PA2072 mutants. Within each group, the 80% confidence ellipses are relatively tight, and biological replicates cluster well, demonstrating a good experimental reproducibility.

Dispersion: Raw read counts were normalized and differential expression analysis was conducted using DEseq2. We have generated MA plots for all key comparisons providing immediate insight into the overall distribution of gene expression and fold-changes, as follows:

MA Plots of Differentially Expressed Genes


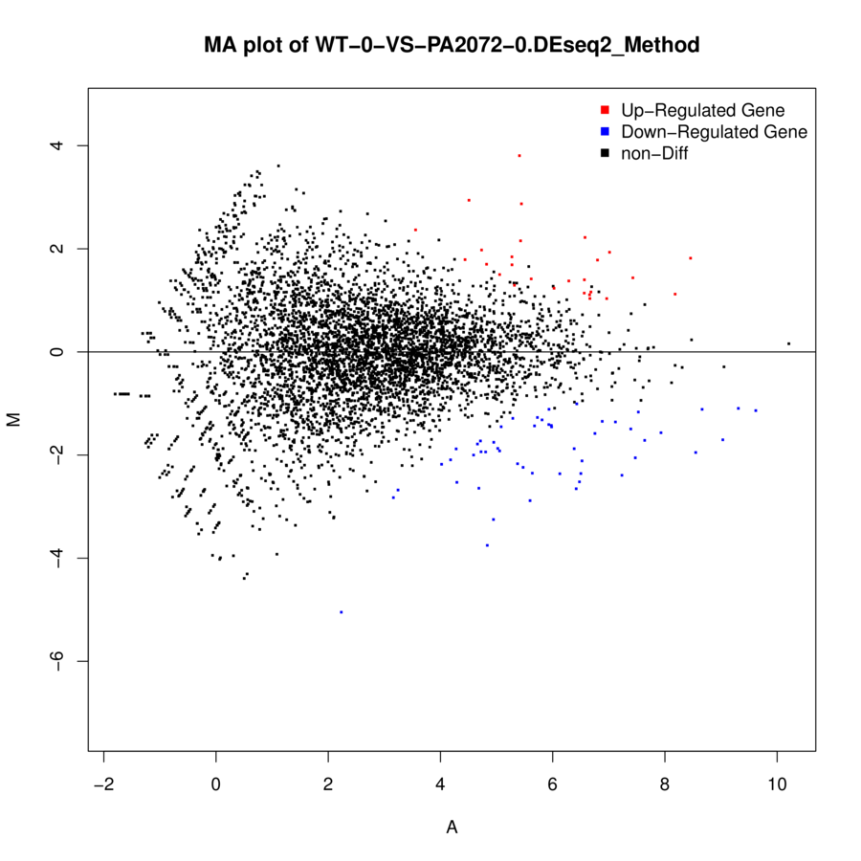


A


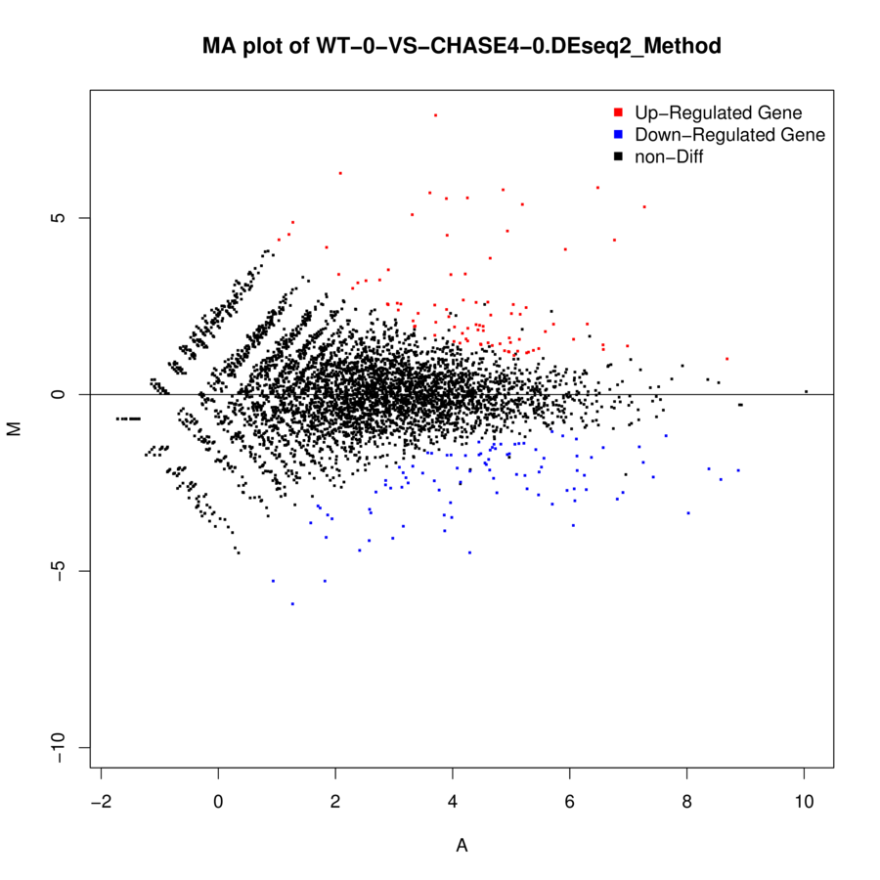


B


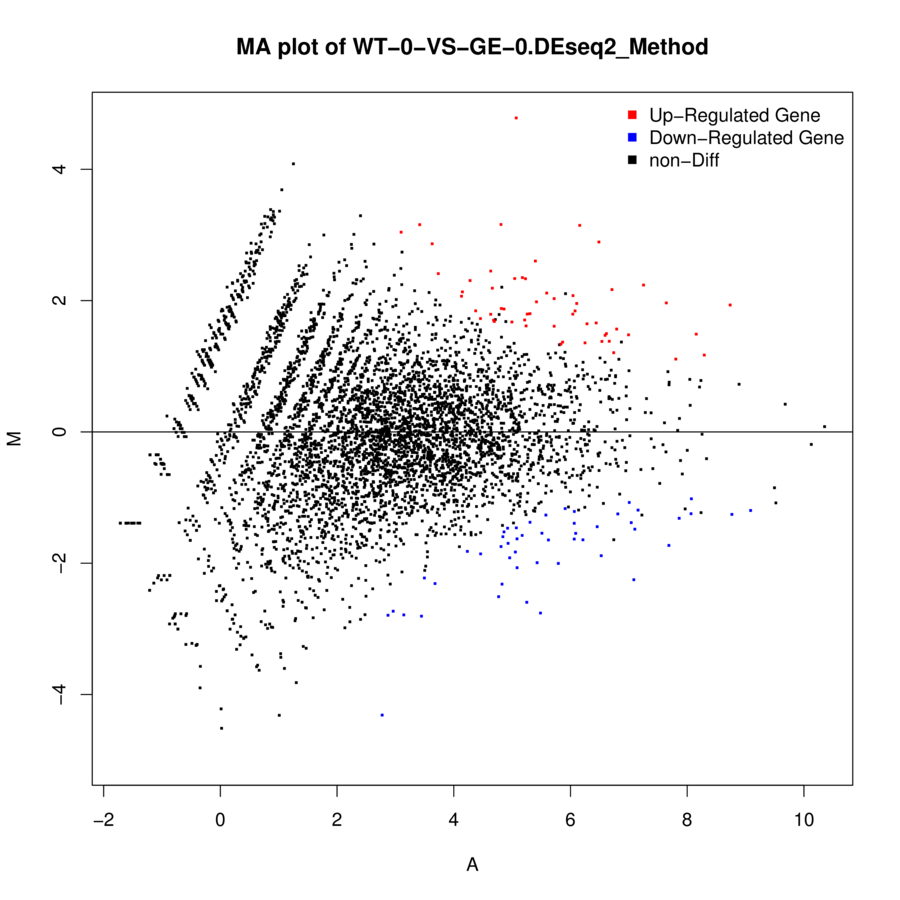


C

Seq Figure 2. MA plots visualizing differentially expressed genes (DEGs) from three pairwise comparisons: *Δpa2072* vs WT(A), *Δpa2072chase4* vs WT(B), *Δpa2072ggdef-eal* vs WT(C), respectively. Differential expression analysis was performed using DESeq2 (n = 3). Significantly upregulated (red dots) and downregulated (blue dots) DEGs are highlighted (|log2(fold change)| ≥ 1, FDR-adjusted *p* value (*p* adj) ≤ 0.05), while non-significant genes are shown in black dots.

Quality Metrics: Statistics on the filtered clean reads, including data volume statistics, base content distribution statistics, and base quality distribution statistics, have been provided as follows:

Seq Table 1. Statistics of Filtered Reads Data Quality Metrics

| Sample | Clean Reads(M) | Clean Bases(G) | Q20(%) | Q30(%) | GC(%) | Read Length(bp) |
| --- | --- | --- | --- | --- | --- | --- |
| *Δpa2072chase4-1* | 9.5989M | 1.4398G | 98.55 | 95.93 | 57.32 | 150 |
| *Δpa2072chase4-2* | 8.6604M | 1.2991G | 98.62 | 96.05 | 57.79 | 150 |
| *Δpa2072chase4-3* | 8.3831M | 1.2575G | 98.70 | 96.25 | 57.96 | 150 |
| *Δpa2072ggdef-eal-1* | 8.0219M | 1.2033G | 99.01 | 96.92 | 58.97 | 150 |
| *Δpa2072ggdef-eal-2* | 7.7580M | 1.1637G | 98.95 | 96.84 | 59.53 | 150 |
| *Δpa2072ggdef-eal-3* | 7.5596M | 1.1339G | 98.96 | 96.83 | 59.24 | 150 |
| *Δpa2072-1* | 7.5391M | 1.1309G | 98.97 | 96.77 | 57.96 | 150 |
| *Δpa2072-2* | 7.4528M | 1.1179G | 98.96 | 96.73 | 58.16 | 150 |
| *Δpa2072-3* | 8.5800M | 1.2870G | 98.92 | 96.70 | 58.66 | 150 |
| WT-1 | 7.9476M | 1.1921G | 98.99 | 96.79 | 57.03 | 150 |
| WT-2 | 7.7612M | 1.1642G | 98.92 | 96.67 | 57.77 | 150 |
| WT-3 | 8.3238M | 1.2486G | 99.00 | 96.85 | 57.54 | 150 |

Volcano Plots: Volcano plots visualizing significance of the differentially expressed genes have been included as follows:

Volcano Plots of Differentially Expressed Genes
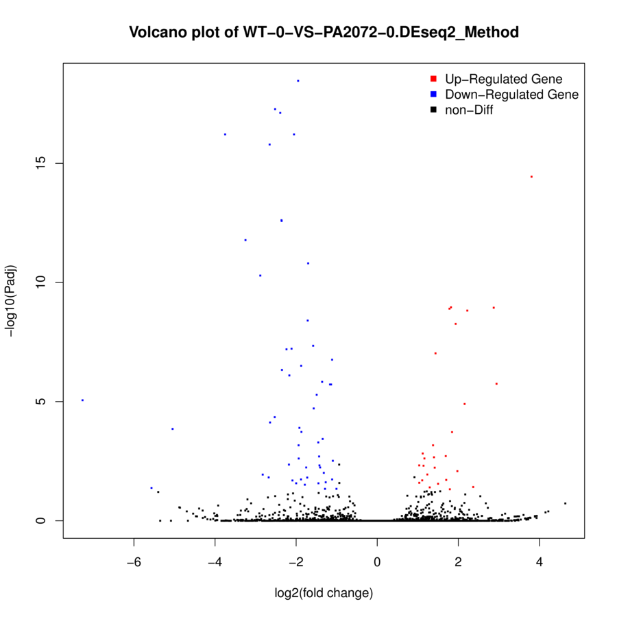


A


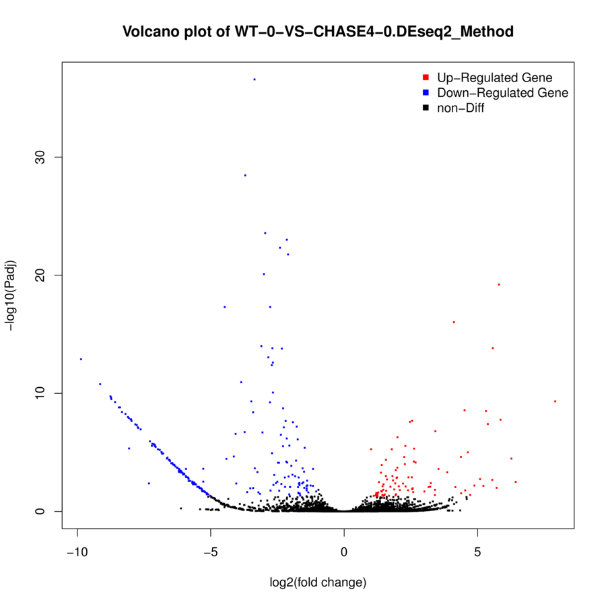

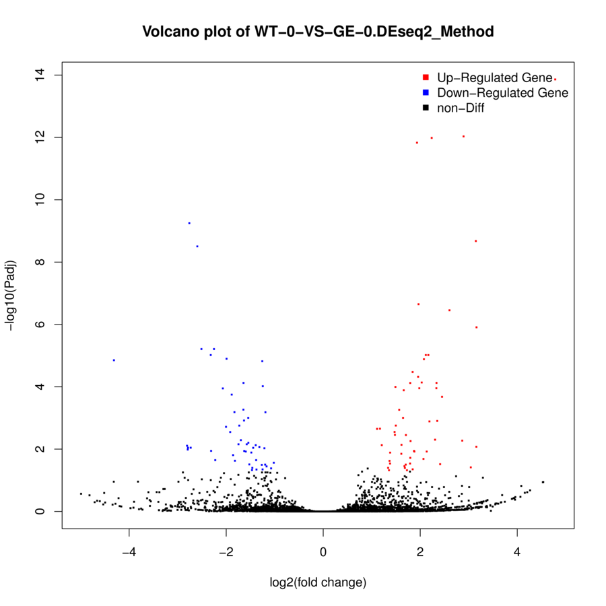


B

C

Seq Figure 3. Volcano plots visualizing differentially expressed genes (DEGs) from three pairwise comparisons: *Δpa2072* vs WT(A), *Δpa2072chase4* vs WT(B)*, Δpa2072ggdef-eal* vs WT(C), respectively. Differential expression analysis was performed using DESeq2 (n = 3). Significantly upregulated (red dots) and downregulated (blue dots) DEGs are highlighted |log2(fold change)| ≥ 1, FDR-adjusted *p* value (*p* adj) ≤ 0.05), while non-significant genes are shown in black dots.

Raw Read Mapping Statistics: We now provide the read mapping statistics below, including the total number of reads per sample and the percentage of reads successfully mapped to the reference genome. This demonstrates the high quality of our sequencing data.

Seq Table 2. Statistics of Read Mapping

| Sample | Total Clean Reads | Total Mapped Reads(%) | Perfect Match(%) |
| --- | --- | --- | --- |
| *Δpa2072chase4-1* | 9589206 | 98.72 | 79.56 |
| *Δpa2072chase4-2* | 8656758 | 98.83 | 80.18 |
| *Δpa2072chase4-3* | 8379928 | 98.84 | 80.33 |
| *Δpa2072ggdef-eal-1* | 8013338 | 95.59 | 78.80 |
| *Δpa2072ggdef-eal-2* | 7749166 | 94.71 | 77.44 |
| *Δpa2072ggdef-eal-3* | 7549460 | 94.10 | 77.17 |
| *Δpa2072-1* | 7530740 | 95.06 | 78.00 |
| *Δpa2072-2* | 7450754 | 95.12 | 78.02 |
| *Δpa2072-3* | 8574374 | 93.10 | 76.03 |
| WT-1 | 7943456 | 97.54 | 80.52 |
| WT-2 | 7757150 | 96.52 | 79.46 |
| WT-3 | 8317210 | 96.45 | 79.56 |
